# Synthetic Biology Approaches for Improving Chemical Production in Cyanobacteria

**DOI:** 10.3389/fbioe.2022.869195

**Published:** 2022-03-11

**Authors:** Tanner R. Treece, Jake N. Gonzales, Joseph R. Pressley, Shota Atsumi

**Affiliations:** ^1^ Department of Chemistry, University of California, Davis, Davis, CA, United States; ^2^ Plant Biology Graduate Group, University of California, Davis, Davis, CA, United States

**Keywords:** CO_2_ fixation, cyanobacteria, photosynthesis, RuBisCO, CRISPR

## Abstract

Biological chemical production has gained traction in recent years as a promising renewable alternative to traditional petrochemical based synthesis. Of particular interest in the field of metabolic engineering are photosynthetic microorganisms capable of sequestering atmospheric carbon dioxide. CO_2_ levels have continued to rise at alarming rates leading to an increasingly uncertain climate. CO_2_ can be sequestered by engineered photosynthetic microorganisms and used for chemical production, representing a renewable production method for valuable chemical commodities such as biofuels, plastics, and food additives. The main challenges in using photosynthetic microorganisms for chemical production stem from the seemingly inherent limitations of carbon fixation and photosynthesis resulting in slower growth and lower average product titers compared to heterotrophic organisms. Recently, there has been an increase in research around improving photosynthetic microorganisms as renewable chemical production hosts. This review will discuss the various efforts to overcome the intrinsic inefficiencies of carbon fixation and photosynthesis, including rewiring carbon fixation and photosynthesis, investigating alternative carbon fixation pathways, installing sugar catabolism to supplement carbon fixation, investigating newly discovered fast growing photosynthetic species, and using new synthetic biology tools such as CRISPR to radically alter metabolism.

## Introduction

It is well established that rising atmospheric CO_2_ levels are the primary cause for unprecedented climate change impacting the globe (Solomon et al., 2009). Despite this, chemical production still relies mostly on petroleum-based synthesis (Levi and Cullen, 2018). In response to the growing concern over greenhouse gasses, research with a focus on more sustainable chemical production has become high priority. The fields of synthetic biology and metabolic engineering aim to achieve a more sustainable method for chemical production using engineered organisms. These efforts include the use of both heterotrophic and photosynthetic microorganisms. Heterotrophic chemical production involves a carbon input of a sugar feedstock to a microorganism to generate a biochemical product as an output. Alternatively, using photosynthetic microorganisms, such as cyanobacteria, offers the advantage of eliminating the need for sugar feedstocks and the ability to generate valuable chemical commodities from CO_2_ and sunlight. The two predominant categories of photosynthetic microorganisms being investigated for chemical production are microalgae and cyanobacteria. Microalgae are a diverse group of photosynthetic eukaryotes that have been shown to be viable production hosts for a wide array of useful chemical commodities ranging from biofuels to lipids and vitamins (Sproles et al., 2021). Cyanobacteria are a group of prokaryotic microorganisms with some of the fastest carboxylation rates present in photosynthetic organisms (Flamholz et al., 2019). The focus of this review will be on recent synthetic biology research in cyanobacteria which have garnered interest as efficient photosynthetic chemical production hosts.

Despite the burgeoning interest around these photosynthetic microorganisms, the field still faces many challenges that have yet to be addressed. The primary concern is the inefficient nature of photosynthesis and CO_2_ fixation. Attempts to improve upon the central carbon fixation enzyme ribulose-1,5-bisphosphate carboxylase-oxygenase (RuBisCO) have been met with little success (Erb and Zarzycki, 2018; Flamholz et al., 2019). Part of the challenge with RuBisCO is its inability to distinguish between CO_2_ and O_2_ with high specificity and notably, the oxygenase activity of RuBisCO results in an energetically costly pathway known as photorespiration which has widespread effects on the growth and metabolic needs of many species of photosynthetic organisms (Hagemann and Bauwe, 2016). Recent research suggests that photorespiration is a symptom of RuBisCO evolving in a high CO_2_ environment where enzymatic specificity was not as vital, with this in mind, recent studies are investigating the possibilities of by reviving ancestral forms of the protein and subjecting it to new environments in the hopes of generating biologically important variants (Shih et al., 2016). Other efforts are looking towards natural adaptations of CO_2_ fixation for inspiration with a focus on the carboxysome, a bacterial microcompartment that acts to localize RuBisCO with high concentrations of CO_2_ (Kerfeld and Melnicki, 2016). These studies aim to avoid photorespiration by engineering synthetic protein structures to mimic cyanobacterial carboxysomes to concentrate CO_2_ near RuBisCO and competitively inhibit the reaction with oxygen (Borden and Savage, 2021). Other efforts to further improve these photosynthetic organisms as chemical production hosts include engineering superior light delivery systems for bioreactors and engineering the light harvesting complexes to take advantage of the entire visible light spectrum (Stephens et al., 2021).

The focus of this review will be on the recent methods employed to overcome the supposed shortcomings of photosynthetic organisms ranging from rewiring carbon metabolism and photosynthesis, introducing additional carbon substrates, generating other chassis organisms capable of superior carbon sequestration, studying faster growing variants of cyanobacteria, and developing new tools via synthetic biology.

### Rewiring Photosynthetic Metabolism

Efforts have been made to overcome the intrinsic shortcomings of photosynthetic microorganisms by rewiring metabolism related to carbon fixation and photosynthesis. While efforts to improve RuBisCO have not been met with much success, current research has shifted focus towards rerouting metabolism to improve overall photosynthetic efficiency by focusing on key aspects of the Calvin-Benson cycle or the photosynthetic electron transport chain (PETC). One strategy used to harness the excess energy being lost by the PETC in cyanobacteria involved overexpressing the protein OmcS (Meng et al., 2021). This strategy coupled the excess electrons from the PETC to NADH production and was shown to increase intracellular ATP and NADH allowing for a fourfold improvement of D-lactate production in the cyanobacterium *Synechococcus elongatus* UTEX 2973 (hereon 2973) (Meng et al., 2021).

An inherent drawback of RuBisCO is its promiscuous nature, when RuBisCO undergoes oxygenase activity a costly side pathway known as photorespiration occurs where the oxygenase product is recycled back into usable metabolism consuming energy and losing CO_2_ in the process. As much as 30% of energy produced by photosynthesis has been observed to be lost through photorespiration in plants (Hagemann and Bauwe, 2016). Rewiring or preventing photorespiration represents a promising way to improve the overall efficiency of carbon fixation in photosynthetic organisms. Efforts to rewire photorespiration generally involve deleting energetically costly steps, circumventing steps where CO_2_ is lost, and rerouting metabolites towards central carbon metabolism (Hagemann and Bauwe, 2016). One of the more ambitious efforts to ameliorate the cost of photorespiration was the expression of a synthetic carbon capture pathway to serve as both a photorespiratory bypass and as a supplement to the Calvin-Benson cycle, this was shown to be a viable use of synthetic biology to counteract the costly natural photorespiration pathway (Shih et al., 2014).

It should be noted that photorespiration is not the sole pathway responsible for carbon inefficiencies, many metabolic processes include steps where CO_2_ is lost to the environment. An important way to engineer microorganisms for sustainability involves carbon conservation, focusing on rerouting metabolism to circumvent decarboxylation reactions (François et al., 2020). Of the more notable strategies is the non-oxidative glycolysis pathway (NOG) which has been shown to function in *Escherichia* coli and which can effectively conserve all carbon associated with sugar catabolism to acetyl-CoA (Bogorad et al., 2013). While carbon conservation is a powerful methodology for engineering metabolism, the field is still in its infancy and further work is required to evaluate the industrial viability of many carbon conservation strategies. Additionally, *de novo* carbon fixation pathways, which will be addressed later in this review, are currently being developed and may prove to be a better methodology for the development of sustainable production hosts.

### Non-RuBisCO Carbon Fixation

In contrast to research centering on canonical CO_2_ fixation, investigations into *de-novo* CO_2_ fixation pathways have been explored and theorized in recent years as more efficient alternatives to traditional RuBisCO based CO_2_ assimilation. These pathways may provide advantages in chemical production hosts by offering insight into carboxylation reactions that could work in tandem with RuBisCO. The expression of formate dehydrogenase in the cyanobacterium, *Anabaena* sp. PCC 7120, was shown to successfully increase intracellular formate concentration, representing an alternative to the photo-reduction of CO_2_ and can act to supplement natural carbon fixation pathways (Ihara et al., 2013).

Many of these *de novo* CO_2_ fixation pathways have had limited success when installed into model organisms such as *E. coli* and yeast and it has yet to be shown if these pathways can function effectively in photosynthetic hosts. One pathway of note that has been shown to work in *E. coli* is the reductive glycine pathway, hereafter RGP (Tashiro et al., 2018). This pathway leverages the native glycine cleavage system in the reverse direction to combine one equivalent of CO_2_ with 5,10-methylenetetrahydrofolate that has been produced from formate to produce pyruvate. This method allows *E. coli* to directly assimilate CO_2_ into central metabolic pathways and is a more efficient method for CO_2_ fixation than traditional RuBisCO (Bar-Even et al., 2013). This inorganic carbon can then be leveraged for biochemical synthesis. Additional work has also recently shown that the expression of formate dehydrogenase confers further renewable characteristics to strains harboring the RGP by removing the need for glucose supplementation (Bang et al., 2020). Other notable CO_2_ fixation pathways include the crotonyl-CoA/ethylmalonyl-CoA/hydroxybutyryl-CoA (CETCH) cycle and the tartronyl-CoA (TaCo) pathways (Scheffen et al., 2021). While the RGP has proven to be a viable carbon fixation pathway that was shown to function in *E. coli,* the growth exhibited by this CO_2_ fixing *E. coli* is slower than its traditional heterotrophic phenotype (Tashiro et al., 2018; Bang et al., 2020). Advancements in modeling and metabolomics may allow for an increase in the creation of *de-novo* carbon fixation pathways that may prove to be both more efficient than traditional pathways and capable of functioning in a wider array of chemical production hosts.

### Photomixotrophy

Another approach to increase chemical production capacity is to supplement CO_2_ with carbohydrates as an auxiliary carbon source for the Calvin-Benson cycle, thereby making the organism photomixotrophic. By re-engineering glucose catabolism to direct carbon flux into the Calvin-Benson cycle, more ribulose-1,5-bisphosphate can be supplied to RuBisCO, accelerating CO_2_ fixation. This ultimately results in faster growth and production of downstream targets, as well as a six-fold increase in titer once metabolism was rewired to accommodate photomixotrophy (Kanno et al., 2017). The addition of a heterotrophic mode also allows for CO_2_ fixation in darkness, resulting in a 24 h production period under natural diurnal conditions. Glucose can be readily obtained from the acid hydrolysis of agricultural waste products such as corn stover, in conjunction with other sugars: xylose, arabinose, and galacturonic acid (Mourtzinis et al., 2016). By installing catabolic pathways for these non-glucose sugars, these agricultural waste products can be used more efficiently. Xylose, the second most abundant sugar in corn stover lysate, has successfully been used to achieve photomixotrophic production of 2,3-butanediol in light and dark conditions with significant improvements in growth and product titer over the equivalent photoautotrophic organism (McEwen et al., 2016). A similar strategy has also recently been used to improve the production of 3-hydroxypropionic acid by 4.1 fold in cyanobacteria through the installation of a xylose photomixotrophic module along with other modifications to help assimilate the additional carbon source (Yao et al., 2022). It should also be noted that the cyanobacterium *Synechocystis* sp. PCC 6803 (hereafter 6803) natively possesses photomixotrophic machinery to assimilate glucose. A recent study was able to improve these photomixotrophic conditions through the installation of the NOG pathway along with targeted gene knockouts to increase the intracellular concentration of acetyl-CoA, thereby improving the growth phenotype of 6803 (Song et al., 2021).

### Discovery of Fast-Growing Cyanobacteria

Most research done in this field focuses on using just a handful of species that have traditionally been used as model organisms to study the mechanics of photosynthesis. With increasing interest in using photosynthetic organisms for industrial production there have been efforts to uncover new species that are faster growing and more receptive to engineering. In the realm of cyanobacterial chemical production, species like 2973 and *S. elongatus* PCC 11802 (hereon 11802) have risen in popularity as they are faster growing than the traditional *S. elongatus* PCC 7942 (hereon 7942) and have been shown to produce higher titers of target chemical products under certain circumstances (Yu et al., 2015; Sengupta et al., 2020). Additionally, these fast-growing organisms are providing inspiration for how to better engineer existing model organisms. The fast-growing cyanobacterium 2973 has relatively little differences genetically when compared to the model cyanobacterium 7942 (Yu et al., 2015). However, a notable difference in 2973 is an increase in the expression levels of PSI, cytochrome b_6_f, and plastocyanin on a per cell basis which improves the downstream flux of electrons from PSII, which helps the faster growing cyanobacteria to better utilize photosynthetic energy (Ungerer et al., 2018). The discovery of new fast-growing cyanobacteria may enhance our understanding of photosynthesis and characterizing the differences between these new species with current model organisms.

### Genome Engineering Tools

In cyanobacteria, traditional genomic modifications are a labor-intensive task and limiting in nature due to the polyploidal nature of these organisms and the need for antibiotic resistance markers (Griese et al., 2011). The current methodology for genomic integration involves constructing a plasmid with an antibiotic selection marker in a plasmid host such as *E. coli.* After introduction of this plasmid to cyanobacteria, several rounds of antibiotic screening are required to ensure complete genome segregation (Golden et al., 1987). This process generally limits the number of modifications that can be performed in a single strain due to the physiological constraints of expressing multiple different antibiotic resistance genes.

The overall task of metabolic engineering in cyanobacteria has been made dramatically more efficient thanks to the advent of CRISPR gene editing which allows for markerless edits (Behler et al., 2018). However, the protein Cas9 is toxic to a number of cyanobacteria species (Wendt et al., 2016). Researchers have recently uncovered other endonucleases that are similarly capable of CRISPR gene editing. The main endonuclease of interest is Cpf1 which, while similar to Cas9, is better tolerated by photosynthetic hosts (Ungerer and Pakrasi, 2016). As the body of research grows around Cpf1, more engineering strategies will be made available in the realm of photosynthetic chemical production and should offer a boon towards the viability of these organisms to begin replacing their non-CO_2_ fixing brethren in the realm of biochemical production (Bishé et al., 2019; Niu et al., 2019). Additionally, having the ability to perform markerless genomic modifications unlocks the potential to engineer these microorganisms far more ambitiously than what was previously possible.

Other work on CRISPR technologies in cyanobacteria includes the use of CRISPR inhibition (CRISPRi) by using dead Cas9 (dCas9) (Qi et al., 2013). While the endonuclease activity of the intact Cas9 protein seems to be toxic to these production hosts, dCas9 is able to function in the same manner in photosynthetic hosts as it is able to in heterotrophic hosts such as *E. coli* (Santos et al., 2021)*.* While the use of dCas9 may not be as broadly useful as traditional CRISPR, dCas9 has been shown to be invaluable in certain chemical production applications where more traditional gene knockouts would otherwise be toxic.

## Concluding Remarks

While many challenges remain and must be overcome to enable widespread adoption of photosynthetic chemical production hosts, the above studies suggest that there are myriad avenues of research that can get closer to this goal. The renewed interest in the field due to the ongoing climate crisis has spurred efforts to improve and adopt these microorganisms as a sustainable alternative for traditional petroleum-based synthesis. Many of the challenges in this field revolve around the intrinsic inefficiencies of carbon fixation and photosynthesis. While engineering RuBisCO remains an interesting target for improving carbon fixation, it has proven to be highly resistant to traditional engineering and decades of research would suggest that it is next to impossible to improve. Focusing on engineered carbon fixation pathways is a more promising route towards improving the carbon sequestration ability of cyanobacteria. Other research into improving the efficiency of photosynthesis by introducing alternative pathways downstream of the PETC for the production of chemical products is a prime example of how we can engineer these microbes to make full use of excess reducing potential from the PETC. The aforementioned approaches aim to enhance our understanding of the inefficiencies related to carbon fixation and photosynthesis while also representing some of the more novel approaches being undertaken by the field of synthetic biology. The discovery of new synthetic biology tools and investigation into faster growing cyanobacteria is also expanding the field of photosynthetic microbial research to make photosynthetic microorganisms a more viable alternative to petroleum based chemical production.

**FIGURE 1:**
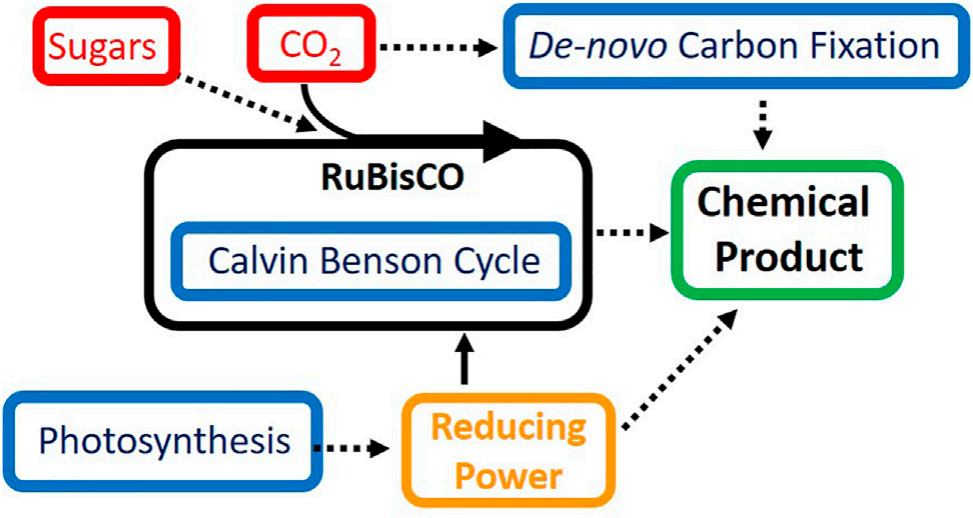
Strategies for improving efficiency of carbon fixation and photosynthesis in cyanobacteria. Shown in red are carbon sources for metabolism in cyanobacteria, in blue are pathways of interest to synthetic biology and metabolic engineering, solid lines represent native pathways and dashed lines represent pathways of interest for improvement using synthetic biology.

Of the discussed challenges for synthetic biology in cyanobacteria, improving the rate and efficiency of carbon fixation seems to be the most difficult, however, this task also holds the most promise. While RuBisCO is resistant to direct engineering strategies, adding additional carbon fixation modules can enhance the viability of cyanobacteria as a chemical production chassis. Further research into *de novo* carbon fixation pathways capable of operating in parallel to the Calvin-Benson cycle and RuBisCO holds great promise for circumventing the inefficiencies of carbon fixation in cyanobacteria. Multiple carbon fixation pathways operating in tandem could exponentially increase the amount of CO_2_ sequestered by cyanobacteria and greatly enhance growth and product formation. The process of carbon fixation is a highly regulated process, and this strategy will likely face further challenges before successful implementation. Overall, it is highly likely that the optimal route for improving the conversion of CO_2_ into valuable chemical commodities in cyanobacteria lies in exploiting multiple of the aforementioned strategies contained within this review (Figure 1).

As new synthetic biology tools become available for cyanobacteria, high throughput screening will allow for rapid progress to be made within this field. The advent of CRISPR technology has had profound effects on research in a wide variety of fields but is relatively new to cyanobacteria. Additionally, faster growing species of cyanobacteria are rising in popularity and more have yet to be discovered. While these newly discovered cyanobacteria are efficient production hosts in their own right, they also inform the field on future targets for modification. Understanding the inherent differences among these organisms is vital to improving our understanding of carbon fixation and photosynthesis. It stands to reason that research into discovering more of these faster growing species, as well as studying the known cyanobacterial variants will provide insight and guidance for future work in this field. While improving photosynthetic production hosts has been historically difficult, the studies described in this work point to a promising future.
